# Cancer stem cell-driven efficacy of trastuzumab (Herceptin): towards a reclassification of clinically HER2-positive breast carcinomas

**DOI:** 10.18632/oncotarget.6094

**Published:** 2015-10-12

**Authors:** Begoña Martin-Castillo, Eugeni Lopez-Bonet, Elisabet Cuyàs, Gemma Viñas, Sonia Pernas, Joan Dorca, Javier A. Menendez

**Affiliations:** ^1^ Unit of Clinical Research, Catalan Institute of Oncology, Girona, Catalonia, Spain; ^2^ Molecular Oncology Group, Girona Biomedical Research Institute (IDIBGI), Girona, Catalonia, Spain; ^3^ Department of Biochemistry and Molecular Biology II, Faculty of Pharmacy, Complutense University, Madrid, Spain; ^4^ Department of Anatomical Pathology, Dr. Josep Trueta Hospital of Girona, Girona, Catalonia, Spain; ^5^ ProCURE (Program Against Cancer Therapeutic Resistance), Metabolism and Cancer Group, Catalan Institute of Oncology, Girona, Catalonia, Spain; ^6^ Department of Medical Oncology, Catalan Institute of Oncology, Girona, Catalonia, Spain; ^7^ Department of Medical Oncology, Breast Unit, Catalan Institute of Oncology-Hospital Universitari de Bellvitge-Bellvitge Research Institute (IDIBELL), L'Hospitalet de Llobregat, Barcelona, Catalonia, Spain

**Keywords:** HER2, cancer stem cells, basal-like, cytokeratins, trastuzumab

## Abstract

Clinically HER2+ (cHER2+) breast cancer (BC) can no longer be considered a single BC disease entity in terms of trastuzumab responsiveness. Here we propose a framework for predicting the response of cHER2+ to trastuzumab that integrates the molecular distinctions of intrinsic BC subtypes with recent knowledge on cancer stem cell (CSC) biology. First, we consider that two interchangeable populations of epithelial-like, aldehyde dehydrogenase (ALDH)-expressing and mesenchymal-like, CD44^+^CD24^−/low^ CSCs can be found in significantly different proportions across all intrinsic BC subtypes. Second, we overlap all the intrinsic subtypes across cHER2+ BC to obtain a continuum of mixed phenotypes in which one extreme exhibits a high identity with ALDH+ CSCs and the other extreme exhibits a high preponderance of CD44^+^CD24^−/low^ CSCs. The differential enrichment of trastuzumab-responsive ALDH+ CSCs *versus* trastuzumab-refractory CD44^+^CD24^−/low^ CSCs can explain both the clinical behavior and the primary efficacy of trastuzumab in each molecular subtype of cHER2+ (i.e., HER2-enriched/cHER2+, luminal A/cHER2+, luminal B/cHER2+, basal/cHER2+, and claudin-low/cHER2+). The intrinsic plasticity determining the epigenetic ability of cHER2+ tumors to switch between epithelial and mesenchymal CSC states will vary across the continuum of mixed phenotypes, thus dictating their intratumoral heterogeneity and, hence, their evolutionary response to trastuzumab. Because CD44^+^CD24^−/low^ mesenchymal-like CSCs distinctively possess a highly endocytic activity, the otherwise irrelevant HER2 can open the door to a type of “Trojan horse” approach by employing antibody-drug conjugates such as T-DM1, which will allow a rapid and CSC-targeted delivery of cytotoxic drugs to therapeutically manage trastuzumab-unresponsive basal/cHER2+ BC. Contrary to the current dichotomous model used clinically, our model proposes that a reclassification of cHER2+ tumors based on the spectrum of molecular BC subtypes might inform on their CSC-determined sensitivity to trastuzumab, thus providing a better delineation of the predictive value of cHER2+ in BC by incorporating CSCs-driven intra-tumor heterogeneity into clinical decisions.

During the past decade, several pathological and immunohistochemical (IHC) sub-classifications have been proposed to better characterize the extensive and heterogeneous molecular features of hormone receptor-positive and triple-negative breast cancer (BC) at the clinical level [1-9]. This type of classification, however, has not been extended to clinically HER2+ (cHER2+) BC. To date, cHER2+ BC, as exclusively determined by immunohistochemistry of HER2 protein overexpression and/or fluorescence *in situ* hybridization of HER2 gene amplification, has been largely considered a single disease entity [10-14]. Presumably, this is due to the apparent dominant role of the HER2 receptor itself on the biology and clinical behavior of HER2+ cells, as well as on the almost universal use of the anti-HER2 monoclonal antibody trastuzumab (Herceptin) to therapeutically manage patients with cHER2+ tumors. Interestingly, the importance of HER2 to distinguish a unique BC subtype might be rather low when compared to the magnitude of the BC genome expression as a whole. In other words, the distinct and intrinsic molecular subtypes (luminal A, luminal B, HER2-enriched [HER2e], basal-like, and claudin-low) appear to retain their biological function and, more importantly, their clinical outcome, regardless of the cHER2+ status [15]. However, although the prognostic value of cHER2+ appears to disappear when the molecular subtype is taken into consideration, little is known about how the co-presence of a given molecular subtype might provide independent predictive information for trastuzumab benefit beyond cHER2+ status.

## THE BASAL-HER2+ SUBTYPE CONFERS THE POOREST BC PROGNOSIS AMONG CHER2+ BCS

We are beginning to appreciate that *de novo* (primary) resistance to trastuzumab might occur inside the framework of a mixed BC subtype, in which HER2 overexpression/amplification takes place within a basal-like molecular background [16-23]. While it is not yet clear which IHC markers (e.g., CK5, CK5/6, CK14, CK17 and/or EGFR), alone or in combination, provide the greatest accuracy in defining basal-like BC, Chung *et al.* [23] have recently described that 37% of 97 patients with stage 1-3 HER2+ BC expressed at least one basal marker. When considering the expression of individual markers, the authors identified 15% of CK5/6+/HER2+, 8% of CK14+/HER2+, and 34% of EGFR+/HER2+. A previous study from the same group reported a basal-HER2+ phenotype in 9% of 131 HER2+ tumors when considering the expression of either CK5/6 or CK14 [19]. In a large series of 713 consecutive hormone receptor-negative invasive BC, Liu *et al.* [17] reported 8% of basal-HER2+ cases expressing HER2 and any of the basal markers CK5/6, CK14, or EGFR. Using a consecutive series of 152 HER2+ primary invasive ductal BC, we recently reported 16% of cHER2+ cases presenting a basal-HER2+ phenotype established solely on expression of the basal marker CK5/6 [22]. Beyond IHC-based sub-classification studies, Prat *et al.* [15] used molecular data derived from DNA, RNA, and protein to determine intrinsic BC subtypes in more than 1,700 patients not treated with trastuzumab. This study confirmed that cHER2+ BC had a 14.1% frequency of the intrinsic basal-like subtype, while a similar likelihood (14.4%) of cHER2+ occurred in intrinsic basal-like subtypes. Interestingly, within cHER2+ tumors, HER2 gene and protein expression was significantly higher not only in the HER2-enriched subtype but also in the basal-like subtype when compared to luminal BC subtypes. All of these studies similarly concluded that basal-HER2+ patients have the worst disease-free and overall survival among all the HER2+ subtypes (i.e., the cHER2+ status does not add independent prognostic value to the intrinsic BC subtype), which was even poorer than that of highly aggressive basal-like BC [17].

## AMONG CHER2+ BCS, A BASAL-LIKE PHENOTYPE PREDICTS THE POOREST PRIMARY RESPONSE TO TRASTUZUMAB

Beyond confirming the notion that the occurrence of a basal-HER2+ phenotype can delineate a subgroup of intrinsically aggressive cHER2+ BC, a recent study by our group was the first to reveal that basal-HER2+ patients might not benefit from the addition of trastuzumab on top of chemotherapy [22]. Accordingly, in the sub-cohort of HER2+ patients (*n* = 69) treated with trastuzumab-based adjuvant/neoadjuvant therapy, the basal-HER2+ phenotype was found to be the sole independent prognostic marker for a significantly inferior time to treatment failure in multivariate analysis. Chung *et al.* [23] have recently confirmed that CK5/6 and EGFR expression are predictive of worse prognosis in HER2+ BC patients treated with trastuzumab. Given the known association between the basal-like subtype with stronger responses to chemotherapy compared with other molecular subtypes, the higher recurrence rates in basal-HER2+ patients receiving adjuvant chemotherapy and trastuzumab should be viewed as the ability of the basal protein expression to dictate *de novo* refractoriness to trastuzumab in cHER2+ patients. Although Chung *et al.* [23] acknowledge that they failed to identify a significant predictive threshold for CK5/6 expression, our study established that a simple CK5/6-based fingerprint using a 10% positivity cutoff might be used as a strong predictive marker of primary refractoriness to trastuzumab [22]. These findings build on the pioneering discoveries in 2007 by Harrys *et al.* [16], who demonstrated that a particular HER2+ tumor phenotype overexpressing genes associated with the basal-like phenotype, including higher expression of basal cytokeratins, exhibited intrinsic resistance to pre-operative trastuzumab and vinorelbine.

## THE BASAL-HER2+ MIXED SUBTYPE ACCUMULATES MULTIPLE CSC-RELATED MECHANISMS OF RESISTANCE TO TRASTUZUMAB

**Figure 1:**
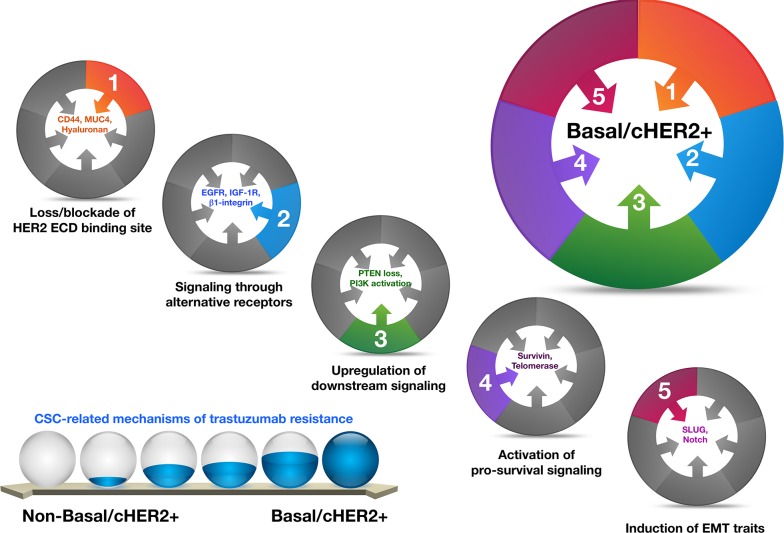
Basal-HER2+ BC cells accumulate a majority of the known mechanisms for trastuzumab resistance, which are not mutually exclusive, and whose key mediators are closely linked to CSCs

There is an ever-expanding body of literature documenting possible mechanisms of escape from trastuzumab that share many of the same markers and signaling pathways implicated in the biology of drug-resistant cancer stem cells (CSCs) [24-44]. Given that enrichment for CSC-like features is a well-known molecular hallmark of highly aggressive basal-like BC [45-57], it is reasonable to propose a causal link between the presence of basal markers (e.g., CK5/6), an augmented CSC activity, and primary resistance to trastuzumab in basal-HER2+ disease. Indeed, earlier studies from our group and others have repeatedly described that basal-HER2+ cells exhibiting *de novo* resistance to trastuzumab distinctively amass a majority of the known mechanisms for trastuzumab resistance [18, 20, 21, 23, 30, 33, 44], which are not mutually exclusive (Figure 1). The key mediators of these mechanisms are closely linked to CSCs [25].

While confirmation of these *in vitro* findings in clinical specimens will be required to unambiguously demonstrate the role of CSCs in clinical resistance to trastuzumab, we should also acknowledge that trastuzumab efficacy appears to rely, at least in part, on its ability to directly target and inhibit CSCs in HER2+ tumors [58-65]. Moreover, HER2 protein itself appears to be a key driver of CSCs in BC even in the absence of HER2 overexpression/amplification [60, 64, 65], which might explain the unexpected clinical efficacy of adjuvant trastuzumab in cHER2-negative BC [66, 67]. Therefore, if the molecular basis for the clinical efficacy of trastuzumab is *via* a CSC targeting-dependent process, the most direct manner to resolve this scenario is the counterintuitive proposal of the *a priori* occurrence of trastuzumab-resistant HER2+ CSCs exclusively in basal-HER2+ BC, but not in other HER2+ phenotypes. That is, “*not all HER2+ CSCs are born equal”,* because basal-HER2+ tumors continue to grow not only when challenged with trastuzumab, but also when treated with other anti-HER therapies (e.g., the dual EGFR/HER2 tyrosine kinase inhibitor lapatinib [30, 43, 68]). Alternatively, when considering the plasticity of CSC-like cellular states in BC [56], perhaps we need to paraphrase Orwell to rather consider that “*all HER2+ CSCs are equal, but some HER2+ CSCs are more equal than others*”. We here propose a framework for predicting the response of cHER2+ to trastuzumab that integrates the molecular distinctions of intrinsic BC subtypes with the most recently acquired data on CSC biology in BC.

## INTRINSIC MOLECULAR SUBTYPES, CSCS STATES AND HER2+ BC: RETHINKING THE PROGNOSTIC-PREDICTIVE VALUE OF CHER2+ TUMORS

Breast CSCs appear to exist in two different, but reversible and therefore interchangeable, epithelial (E)- and mesenchymal (M)-like states [56]; the first state characterized by the expression of aldehyde dehydrogenase (ALDH) [58, 69-73], and the second state characterized by the CD44^+^CD24^−/low^ immunophenotype [74-76]. Importantly, while these distinct CSC populations can be found across all the molecular/intrinsic BC subtypes, their proportion varies significantly. Accordingly, the number of ALDH-expressing E-CSCs is significantly higher in HER2-enriched (HER2e) and luminal B BC, while basal-like and claudin-low BC are highly enriched in CD44^+^CD24^−/low^ M-CSCs. Moreover, the gene expression profiles of E-CSCs resemble those of luminal stem cells, whereas the profiles of M-CSCs resemble those of basal stem cells in normal breast [56, 77-79]. Given this knowledge and the understanding that different BC molecular subtypes are characterized by distinct mutational portraits [80], a unique combination of genetic and likely also microenvironmental factors will ultimately contribute to the predominance of each CSC phenotype in a given cHER2+ tumor type.

**Figure 2:**
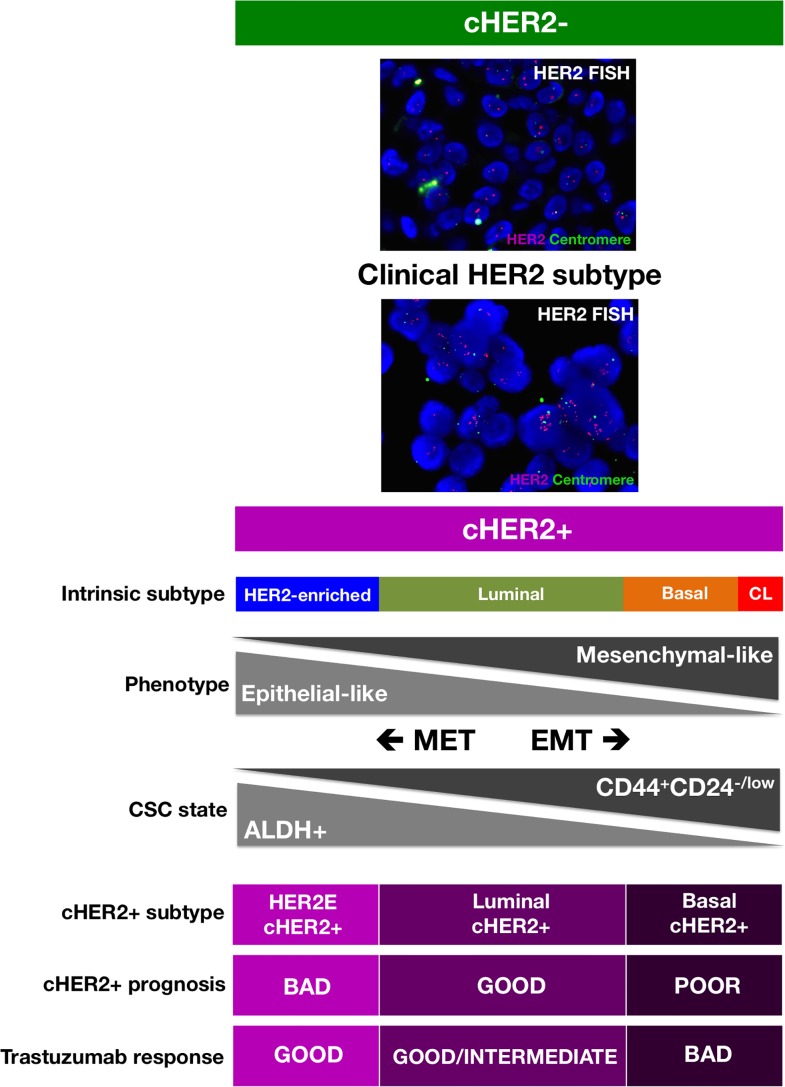
A new framewok for predicting the response of cHER2+ to trastuzumab that integrates the molecular distinctions of intrinsic BC subtypes with the most recent knowledge of CSC biology (CL: Claudin-low; HER2E: HER2-enriched; MET: Mesenchymal-Epithelial Transition; EMT: Epithelial-Mesenchymal Transition).

We propose that when overlapping each molecular BC subtype across the continuum of cHER2+, where one extreme is the complete absence of CD44^+^CD24^−/low^ M-CSCs in HER2-enriched/cHER2+ tumors and the other extreme is a high preponderance of CD44^+^CD24^−/low^ M-CSCs in basal/cHER2+ and claudin-low/cHER2+ tumors, the differential enrichment of ALDH-expressing *versus* CD44^+^CD24^−/low^ CSCs might explain both the clinical behavior and the primary efficacy of trastuzumab in each mixed cHER2+ subtype (Figure 2). On the one hand, the enrichment of ALDH-expressing E-CSCs in HER2e/cHER2+ and luminal B/cHER2+ subtypes might contribute to the known poor clinical outcome of BC co-overexpressing ALDH and HER2 [81, 82]. Simultaneously, given that efficacy of trastuzumab appears to relate directly to its ability to drastically reduce the fraction of ALDH-positive cells in HER2+ BC cell populations (i.e., CSC populations defined by high ALDH express the highest HER2 levels and remain exquisitely sensitive to trastuzumab treatment, whereas HER2+ BC cell populations exhibiting primary resistance to trastuzumab maintain an intact population of ALDH+ cells following trastuzumab treatment [58]), HER2e/cHER2+ and luminal B/cHER2+ BC will be the mixed phenotype which will benefit greatest from trastuzumab (Figure 2). If the highest preponderance of ALDH-expressing E-CSCs occurs in the HER2e/cHER2+ and luminal B/cHER2+ BC subtypes and not in the luminal A/cHER2+ subtype, this model could also explain why a minority of patients with cHER2+ BC have an excellent prognosis even in the absence of treatment [83, 84]. On the other hand, the enrichment of CD44^+^CD24^−/low^ M-CSCs may contribute to the highly aggressive clinical behavior of the basal/cHER2+ phenotype since CD44^+^CD24^−/low^ cells will endow this subtype with a mesenchymal-related enhancement of BC progression [85-87]. Simultaneously, since the enrichment of mesenchymal traits results in a highly refractory response to the anti-tumor actions of trastuzumab [20, 21, 30, 33], the basal/cHER2+ and claudin-low/cHER2+ BC will be the phenotypes with less benefit from this treatment.

## INTERCHANGEABLE EPITHELIAL/MESENCHYMAL CSC CELLULAR STATES AND TRASTUZUMAB RESISTANCE: PREDICTING THE CLINICAL BEHAVIOR OF CHER2+

Our current framework of CSC-driven primary efficacy of trastuzumab in cHER2+ also incorporates the idea that breast CSCs have an intrinsic plasticity allowing the transition between the epithelial-like (ALDH+) state and the mesenchymal-like (CD44^+^CD24^−/low^) state. These reversible transitions might endow cHER2+ tumors with a plastic capacity for tissue invasion, dissemination, and growth at metastatic sites, thus ultimately determining the prognosis of each mixed cHER2+ subtype. Alternatively, the ability of CSCs to transit from a trastuzumab-responsive epithelial-like state to a trastuzumab-refractory mesenchymal-like state might endow cHER2+ with a plastic degree of responsiveness to trastuzumab, thus ultimately determining the predictive value of each cHER2+ subtype. Once again, however, it should be noted that the degree of plasticity that enables CSCs (and non-CSC) to transit between these states should vary among each molecular subtype of cHER2+: cHER2+ BC of luminal origin will be more refractory, but still capable, for loss of an epithelial-like state, whereas basal/cHER2+ and claudin-low/cHER2+ BC will be inherently poised to gain a mesenchymal-like state. Indeed, our proposed model can readily accommodate the current available data regarding the occurrence of trastuzumab refractoriness driven by the epithelial-to-mesenchymal transition (EMT) phenomenon [20, 21, 34, 88-92].

JIMT-1 was the first commercial cell line established from a HER2+ patient with intrinsic resistance to trastuzumab, and is naturally enriched for a CD44^+^CD24^−/low^ mesenchymal state. Interestingly, the subpopulation of trastuzumab-refractory basal/HER2+ JIMT-1 cells exhibiting CD44^+^CD24^−/low^ M-CSC-like surface markers changes over time [30]. Whereas low-passage cultures contain ≈10% of cells with the CD44^+^CD24^−/low^ immunophenotype, late-passage JIMT-1 cultures accumulate ≈80% of CD44^+^CD24^−/low^ cells, thus exhibiting an almost perfect identity to the CD44^+^CD24^−/low^-enriched phenotype constitutively occurring in the commonly HER2-negative claudin-low subtype [90-92]. Resistance to trastuzumab (and lapatinib) has been shown to occur when HER2+ cells spontaneously switch from a luminal to a claudin-low phenotype following EMT [93]. The natural ability to acquire a trastuzumab-refractory EMT phenotype might however be higher in HER2+ cells lacking some key epithelial markers such as estrogen receptor and E-cadherin, and are therefore poised to acquire a new mesenchymal state. In contrast, HER2+ cells expressing high levels of markers typical of the luminal phenotype will maintain their trastuzumab-responsive epithelial-like phenotype. Thus, when contemplating a continuum of epithelial-like and mesenchymal-like CSC states throughout the overlapping molecular BC subtypes in cHER2+, it becomes obvious that the EMT phenomenon is a pivotal mechanism that, when activated, convergently drives primary and secondary resistance to HER2-targeted therapies (for a more detailed explanation of how the EMT phenomenon impacts intra-tumor heterogeneity and trastuzumab efficacy in cHER2+ BC, see BOX1).

**Figure 3:**
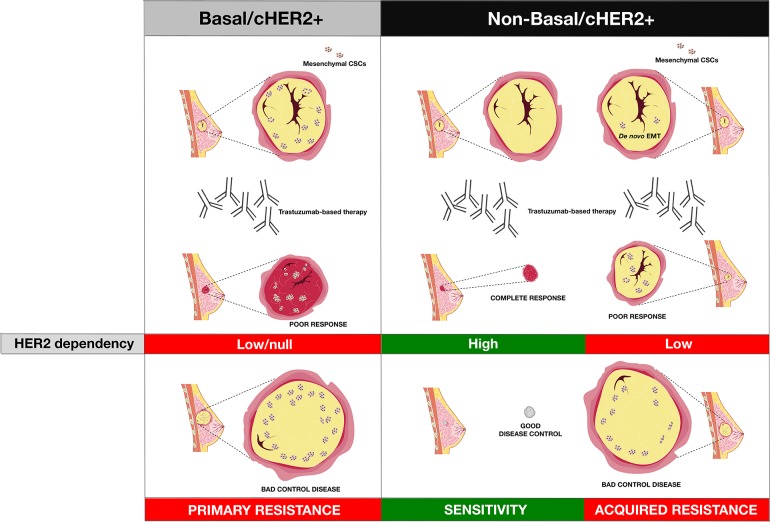
The intrinsic degree of plasticity determining the ability of cHER2+ BC to switch between epithelial and mesenchymal CSC states will vary across the continuum of mixed phenotypes, thus dictating their evolutionary response to trastuzumab

It is noteworthy that the *de novo* enrichment of EMT traits, which appears to be a major determinant of primary resistance to trastuzumab in basal/cHER2+ BC cells, and the spontaneous acquisition of EMT traits, which may constitute a major determinant of acquired resistance to trastuzumab in HER2e and luminal (A and B)/cHER2+ BC cells, both converge on a significant decrease in HER2 expression. Our work showed that the spontaneous enrichment of the CD44^+^/CD24^−/low^ CSC-mesenchymal phenotype in basal/HER2+ cells was coincidental with a global decrease in HER2 expression [30]. Using luminal-HER2+ BC cells, Lesniak *et al*. [93] reported that the spontaneous conversion of trastuzumab-responsive luminal/HER2+ cells to a trastuzumab-refractory CD44^+^/CD24^−/low^ phenotype through EMT was accompanied by a strong down-regulation of HER2. These findings may have major clinical implications when considering the discordance rates for HER2 expression between matching and metastatic tumors [94], and following trastuzumab-based neoadjuvant systemic therapy [95, 96]. Notably, cHER2+ patients whose metastatic disease has converted to HER2-negative have a worse overall prognosis [97], while cHER2+ patients whose residual disease following treatment with neoadjuvant trastuzumab abrogates HER2 overexpression have a significantly poorer recurrence-free survival (RFS) [98]. If a change in HER2 status merely reflects the heterogeneity of HER2 expression within the tumor (i.e., trastuzumab treatment eliminates HER2-overexpressing clones leaving only HER2-negative tumor cells upon completion of therapy), it then follows that trastuzumab should have an equal effect on those tumors achieving a pathological complete response and those tumors becoming HER2-negative. However, the fact that the RFS is significantly better for cHER2+ patients with tumors that retain HER2 overexpression implies that negativization of HER2 is accompanied by an increased aggressiveness in residual disease. Basal/cHER2+ tumors rarely exhibit a uniformly positive basal cytokeratin expression, but instead show a partially-positive type (“baso-luminal” [99]) often displaying a checkerboard-type intratumoral heterogeneity [18]. Therefore, an enrichment of clones or cell clusters with a high percentage of CSCs displaying a basal/mesenchymal phenotype and decreased HER2 expression might explain the poor response of basal/cHER2+ to trastuzumab, as well as the change in HER2 expression status (Figure 3). If a major determinant of trastuzumab resistance in luminal and HER2e/cHER2+ tumors is the *de novo* occurrence of EMT phenomena, leading to the appearance of mesenchymal clones or cell clusters with a CD44^+^/CD24^−/low^/HER2-low phenotype, the selection pressure of trastuzumab treatment will similarly lead to the emergence of trastuzumab-refractory mesenchymal-CSCs, as well as a shift in the HER2 status of the tumor (Figure 3, BOX1). Indeed, the plasticity between CSC types ultimately will result in significant challenges not only to the efficacy of trastuzumab itself but also to trastuzumab-based chemotherapy. EMT phenotypic shifts resulting in increased numbers of M-CSCs will increase the local recurrence capacity of a given cHER2+ subtype by decreasing proliferation and thus generally avoiding the activity of cytotoxic chemotherapeutic agents. By increasing the proportion of trastuzumab- and chemotherapy-refractory low-proliferative/quiescent M-CSCs cells at the invasive edge, a cHER2+ tumor belonging to a given molecular subtype would also augment its capacity of entering the circulation and forming micro-metastases at distant sites. Genetic diversity, epigenetic activation of signaling pathways, and the tumor microenvironment will influence, independently or simultaneously, the plastic capacity of M-CSCs to transitioning back to a proliferative state driven by E-CSCs after cessation of treatment, thus mediating local and metastatic tumor relapses over time (BOX 1).

Forthcoming clinical studies should clarify whether intratumoral heterogeneity in basal cytokeratins and/or conventional EMT markers can confirm a crucial role of HER2-negative M-CSCs in determining trastuzumab efficacy and patient survival in cHER2+ BC. Available *in vitro* data, however, appears to confirm that the degree of intrinsic plasticity to drive the trastuzumab-refractory CD44^+^/CD24^−/low^ mesenchymal CSC state may account for the *de novo* resistance to trastuzumab in basal/cHER2+ BC. Experiments in our laboratory have shown that trastuzumab-refractory basal/HER2+ JIMT-1 cells can be converted into trastuzumab-responsive cells by promoting the conversion of CD44^+^/CD24^−/low^ M-CSC-like states into CD24+ E-CSC-like CSC states [20]. Indeed, the depletion of SLUG, a critical regulator of epithelial cell identity in breast development and cancer [100], was sufficient to drastically reduce the percentage of cells with a trastuzumab-refractory CD44^+^/CD24^−/low^ immunophenotype. Moreover, this sensitized the original basal-HER2+ cell population by increasing the number of trastuzumab-responsive ALDH-expressing cells (unpublished observations). The fact that metformin-induced preferential killing of CD44^+^/CD24^−/low^ cells was similarly sufficient to overcome primary resistance to trastuzumab in basal/HER2+ xenografts *in vivo* [33], lends pharmacological support to the concept that the relative enrichment in CD44^+^/CD24^−/low^ M-CSCs is a/the key determinant of the *de novo* efficacy of trastuzumab. Accordingly, diverse molecular mechanisms leading to the reversal of the mesenchymal phenotype in HER2+ tumors (e.g., mIR-200c-driven suppression of TGF-β signaling [101, 102]) have been found to efficiently counteract trastuzumab resistance and the invasion-metastasis cascade in cHER2+ BC.

## CSC-DRIVEN EFFICACY OF TRASTUZUMAB AND THE RECLASSIFICATION OF CHER2+ BC: A THERAPEUTIC COROLLARY

Despite the undeniable improvements in treatment, many cHER2+ BC patients ultimately die because of disease progression. It is thus critical to increase success rates in the adjuvant/neoadjuvant setting. Unfortunately, no clear biomarkers have emerged as reliable predictors of primary resistance to trastuzumab. Although newer agents are under investigation and are expected to improve outcomes for early-stage patients in combination with trastuzumab-based adjuvant therapy, at the moment we cannot offer clinicians clear guidance on solutions that can be integrated in the daily clinical routine. Because the biologically-distinct intrinsic BC subtypes appear to retain their individual molecular characteristics and biological behavior regardless of cHER2+ status, prospective studies are needed to test the concept that the overlap of each molecular BC subtype across the cHER2+ BC spectrum might generate an *a priori* prediction model of response to trastuzumab. In this framework, the known aggressiveness of the basal-like BC subtype confers not only worsened outcomes for patients with cHER2 BC, but also the poorest response to trastuzumab. Contrary to the current dichotomous clinical model (cHER2+ *versus* cHER2-), our model proposes that reclassification of cHER2+ tumors based on CSC-related markers might indirectly inform on their CSC-driven refractoriness to trastuzumab, thus providing a better delineation of the predictive value of cHER2+ in BC (BOX 1). Indeed, an equivalent overlapping of the intrinsic BC subtypes across the continuum of cHER2-negative BC can explain the apparently paradoxical activity of trastuzumab in HER2e/cHER2− and luminal/HER2− tumors [66, 67], which accumulate HER2-dependent (but not due to gene amplification) ALDH-overexpressing E-CSCs. This can also explain the inefficiency of trastuzumab in basal/cHER2− tumors, which accumulate HER2-independent CD44^+^/CD24^−/low^ M-CSCs [64, 65]. Because only strongly ALDH-positive cells show a more aggressive phenotype typical of E-CSCs [103], it might relevant to evaluate whether the stem cell biomarker ALDH could be associated with trastuzumab efficacy in a cut-off-dependent manner in cHER2+ *versus* cHER2− BC. Nevertheless, it is important to stress that at least two key questions need to be answered before a mixed molecular/clinical classification can be implemented to aid oncologists in the therapeutic management of HER2+ tumors.

First, it is important to address whether the plastic transition between trastuzumab-responsive epithelial-like CSC states and trastuzumab-refractory mesenchymal-like CSC states can similarly explain the efficacy of trastuzumab in the metastatic setting. Although Giordano *et al*. [104] reported that patients with HER2+ metastatic BC have circulating tumor cells (CTCs) undergoing EMT and enrichment for CSC features, additional studies are needed to determine whether EMT-CTCs [105-107] and CSCs have prognostic or predictive value in HER2+ metastatic BC treated with trastuzumab-based therapy. Interestingly, overexpression of the putative CSC biomarker beta1-integrin, a structural component of basal epithelial cells, is an independent negative prognostic factor for tumor progression of HER2+ metastatic BC treated with trastuzumab-based chemotherapy [108]. Beta1-integrin is constitutively overexpressed in basal/HER2+ BC cells with *de novo* resistance to trastuzumab [21, 109], whereas the expression of a heavily N-glycosylated variant of beta1-integrin is activated during the spontaneous conversion of trastuzumab-sensitive HER2+ luminal cells to a trastuzumab-unresponsive HER2− claudin-low phenotype. Our current framework therefore predicts that a primary basal/cHER2+ or claudin-low/cHER2+ phenotype would likely remain unchanged in metastatic disease, whereas one should expect a higher degree of phenotypic conversion during the metastatic evolution of primary luminal/cHER2+ phenotypes.

**Figure 4:**
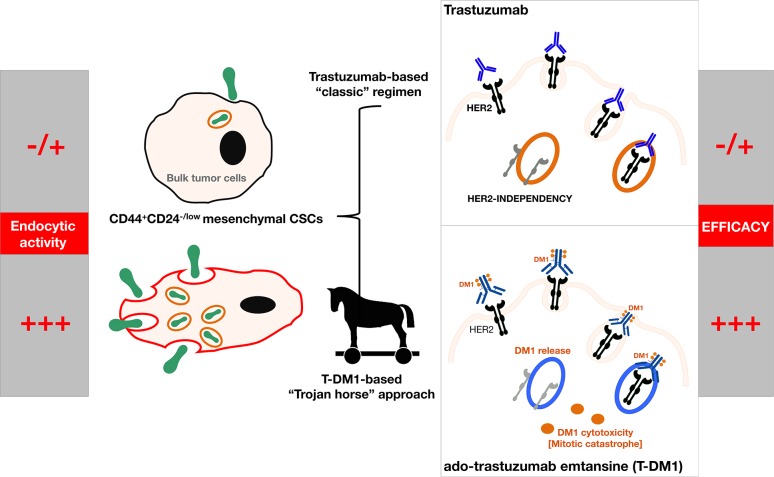
Because trastuzumab-refractory CD44+CD24^−/low^ mesenchymal-like CSCs distinctively possess a highly endocytic activity, the otherwise irrelevant HER2 in basal/cHER2+ can open the door to a “Trojan horse” type approach by employing antibody-drug conjugates such as T-DM1, which will allow a rapid and CSC-targeted delivery of cytotoxic drugs to therapeutically manage cHER2+ BC with primary resistance to trastuzumab

Second, a definition of potentially clinically actionable groupings of cHER2+ BC that improves prognosis and therapeutic planning, especially in the sub-class of basal/cHER2+ and claudin-low/cHER2+ BC patients that are not likely to benefit from trastuzumab-based therapy, should be accompanied by alternative therapeutics able to eliminate the clinically-critical tumor cell population of trastuzumab-unresponsive CSC mesenchymal states. The ability of the anti-diabetic drug metformin to suppress self-renewal and proliferation of trastuzumab-resistant CSCs [33, 110, 111] is under evaluation in the METTEN study, a phase II, randomized, open-label, multicentric trial of neo-adjuvant chemotherapy and trastuzumab with or without metformin in women diagnosed with HER2-positive primary BC [112]. Interestingly, the new antibody-drug conjugate ado-trastuzumab emtansine (T-DM1, Kadcyla^®^), which consists of the potent chemotherapeutic DM1 (maytansinoid) coupled to trastuzumab, has been shown to potently and differentially target trastuzumab-refractory mesenchymal CSCs [113]. It appears that CD44^+^/CD24^−/low^ cells display a highly endocytic activity, thereby rendering them particularly sensitive to antibody-drug conjugates such as T-DM1. Indeed, treatment with T-DM1 not only depleted pre-existing CD44^+^/CD24^−/low^ cells at concentrations that failed to affect the bulk of tumor cells, but also prevented the EMT-mediated induction of CSC-like properties in differentiated tumor cells [113]. The unanticipated targeting of the mesenchymal state of CSCs by T-DM1 may indeed explain the efficacy of this recently approved antibody-drug conjugate against the outgrowth of trastuzumab-refractory basal/HER2+ BC cells xenotransplanted in animal models [114]. Specifically, because CD44^+^CD24^−/low^ mesenchymal-like CSCs distinctively possess a highly endocytic activity, the otherwise irrelevant HER2 can open the door to a “Trojan horse” type approach through the employment antibody-drug conjugates such as T-DM1, which will allow a rapid and CSC-targeted delivery of cytotoxic drugs to trastuzumab-unresponsive basal/cHER2+ BC (Figure 4).

**Figure 5:**
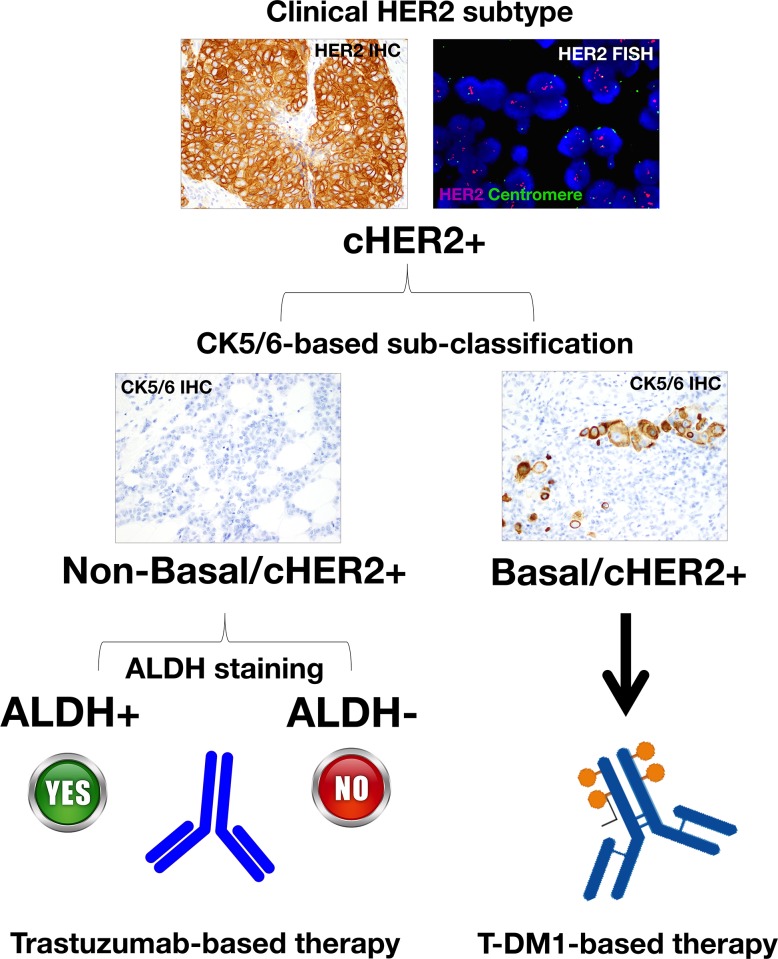
A reclassification of cHER2+ tumors based on basal **(e.g.,** cytokeratin 5/6) and CSC-related markers (e.g., ALDH) might inform on their CSC-determined sensitivity to trastuzumab and T-DM1, thus providing a better delineation of the predictive value of cHER2+ in BC.

The extension of BC subtypes in clinical settings such as triple-negative BC could lead to “orphan” BC diseases that might complicate the accurate design of powerful clinical trials with sufficient number of patients. In contrast, the integration of a straightforward and inexpensive IHC-based subclassification of cHER2+ BC into “basal-like/cHER2+” and “non-basal/cHER2+” subtypes is likely to provide better-quality prognostic and predictive information that might streamline translational medicine for the treating oncologist. T-DM1 has received regulatory approval for treatment-refractory HER2+ metastatic or locally advanced BC. Therefore, if T-DM1 or other new CSC-targeting agents now entering clinical trials might improve clinical outcomes for patients with trastuzumab-unresponsive basal/cHER2+ BC, the reclassification of cHER2 tumors based on basal and CSC-related markers (Figure 5) will undoubtedly lead to further gains for women diagnosed with cHER2+ BC disease. Nevertheless, our model proposes that a reclassification of cHER2+ tumors based on the spectrum of molecular BC subtypes might inform on their CSC-determined sensitivity to trastuzumab at the level of individual tumors (BOX1), thus providing a better delineation of the predictive value of cHER2+ in BC by incorporating CSC-driven intra-tumor heterogeneity into clinical decisions.

BOX 1 **CSC-DRIVEN TUMOR HETEROGENEITY AND TRASTUZUMAB RESISTANCE IN CHER2+ BC**

One of the greatest challenges for BC therapy is the occurrence of intra-tumor cellular heterogeneity [115-118], which negatively affects the efficacy of different approaches to cancer treatment including biological agents such as the anti-HER2 monoclonal antibody trastuzumab (Herceptin). Although the mechanisms driving intratumoral variation of cellular function have remained uncertain until recently, we are accumulating ever-growing evidence that genetic diversity, epigenetics, and the tumor microenvironment ultimately determine the integrated functioning of molecular programs that govern and maintain CSCs across the diverse molecular subtypes of BC [119, 120]. In our current CSC-based framework of primary resistance to trastuzumab in cHER2+ BC, genetic (i.e., mutational landscape) and non-genetic (i.e., epigenetic and microenvironmental) mechanisms collectively but differentially contribute to tumor heterogeneity of cHER2+ tumors belonging to each molecular subtype of cHER2+ BC (i.e., luminal A/cHER2+, luminal B/cHER2+, HER2-enriched/cHER2+, basal/cHER2+, and claudin-low/cHER2+; Figure B1-1).

**Figure B1-1:**
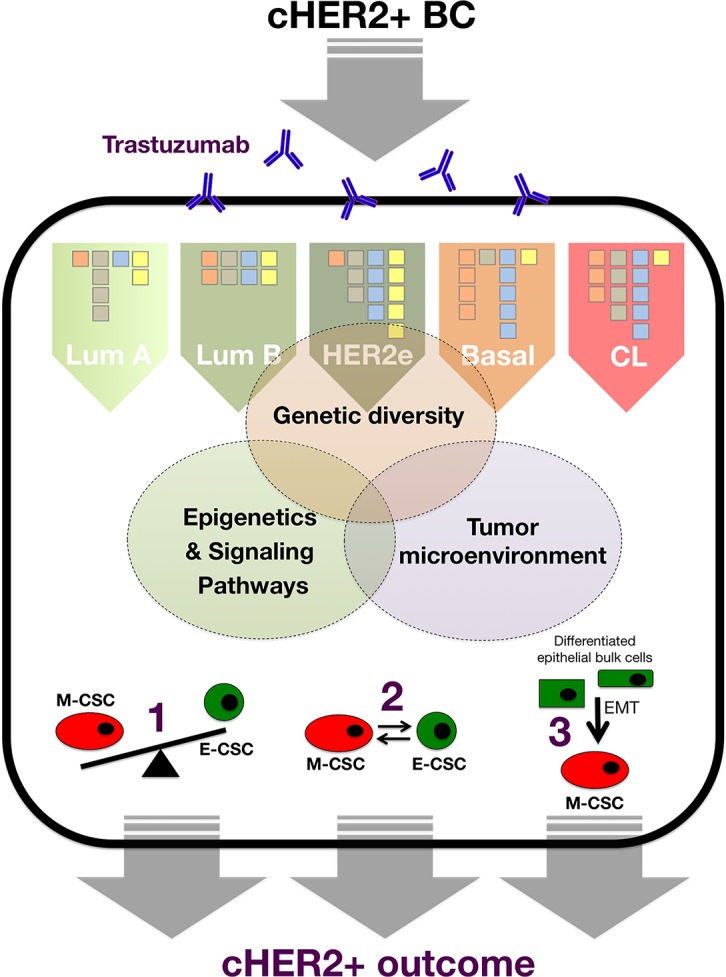
The clinical relevance of each genetic, epigenetic, and microenvironmental facet in terms of trastuzumab failure and recurrence of individual cHER2+ tumors will be related to the extent to which each mechanism, across the spectrum of cHER2+ BC molecular subtypes, impinges on intra-tumor cellular heterogeneity via 1.) the frequency of the trastuzumab-responsive epithelial-CSC type the versus trastuzumab-resistant mesenchymal-CSC type, 2.) the intrinsic differential plasticity that mediates the balance between the two CSC states, and 3.) the intrinsic proclivity of bulk tumor cells to dedifferentiate and acquire trastuzumab-resistant mesenchymal-CSC-like states.

## SOURCES OF CSC-RELATED TUMOR HETEROGENEITY IN CHER2+ BC

### Cellular origin and mutation profile across the spectrum of cHER2+ BC

Each molecular subtype of cHER2+ BC has a different cell-of-origin (e.g., mammary stem cells, bipotent progenitors, luminal progenitors, late luminal progenitors or more committed, differentiated luminal cells), which may represent a stage of developmental arrest for a cHER2+ tumor with an origin earlier in the differentiation hierarchy or, alternatively, transformation of a cell type at one specific stage of development in the normal breast tissue [91, 121, 122]. In addition to having different cells of origin, the different molecular subtypes of cHER2+ BC are characterized by different mutational profiles. Thus, beyond the overexpression/amplification of HER2 shared among all of them, each of the molecular subtypes of cHER2+ BC have a different mutational landscape [123-126], e.g., whereas the most frequent genetic alteration expected to be found in luminal A/cHER2+ BC is the mutational activation of PI3K signaling, basal/cHER2+ and claudin-low/cHER2+ BC subtypes almost always contain mutations in TP53 as well as genomic alterations in PTEN (Figure B1-2).

**Figure B1-2:**
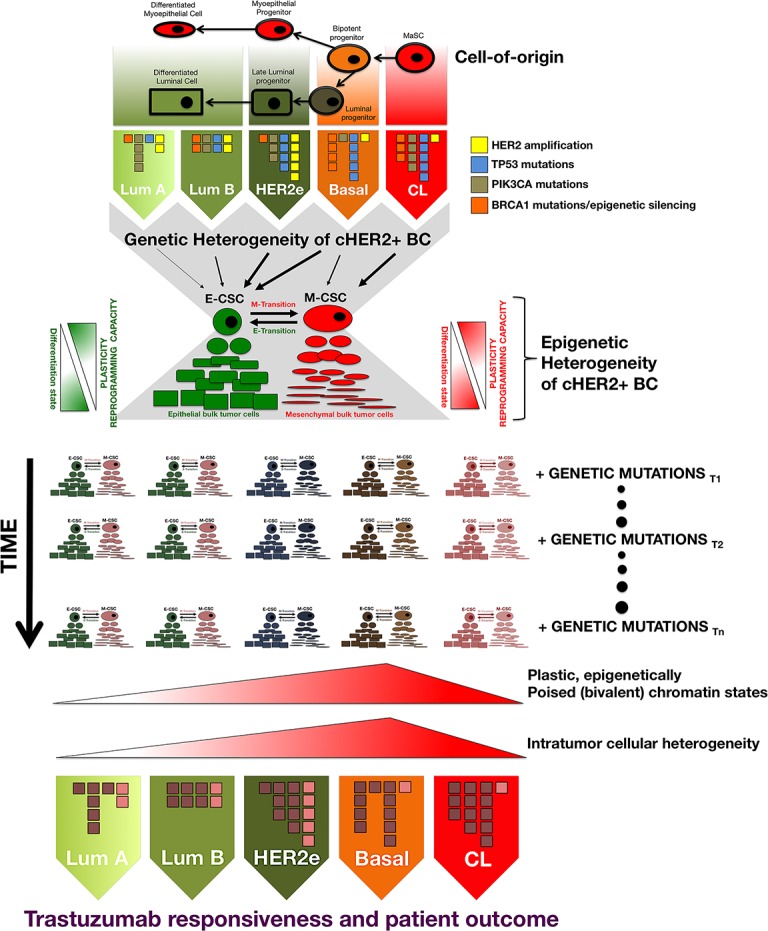
Genetic and epigenetic heterogeneity coalesce at the CSC level to differentially affect tumor evolution and clinical progression in individual tumors belonging to each cHER2+ molecular subtype CSC states may serve as the unit of selection in the genetic evolution of individual cHER2+ tumors belonging to luminal A/cHER2+, luminal B/cHER2+, HER2-enriched/cHER2+, basal/cHER2+, and claudin-low/cHER2+ subtypes because genetic and non-genetic mechanisms can influence CSC properties by acting not only simultaneously but also independently over time, thereby differentially influencing trastuzumab responsiveness, tumor progression, and patient survival in each mixed subtype of cHER2+ BC.

### Variations in trastuzumab-responsive versus trastuzumab-refractory CSC types across the spectrum of cHER2+ BC

Beyond the mutation profile as a source of genetic heterogeneity in the distinct molecular subtypes of cHER2+ tumors, another level of epigenetic heterogeneity arises from the nature of the cells that are responsible for tumor maintenance and progression in each cHER2+ BC subtype, i.e., the so-called CSCs. Despite the diversity of genetic changes driving the different molecular subtypes, two different types of CSCs exist in any of the cHER2+ BC subtypes: a more proliferative, epithelial-like state characterized by the expression of the CSC marker ALDH, and a more quiescent and invasive, mesenchymal-like state characterized by the expression of the CD44^+^CD24^−/low^ immunophenotype [56, 120] (Figure B1-2). This apparently paradoxical phenomenon is suggestive of common, shared regulatory pathways capable of directing self-renewal and differentiation of solely two major types of CSCs regardless of the intrinsic molecular BC subtype in which they reside. Remarkably, however, each molecular subtype of cHER2+ BC is expected to exhibit significantly different frequencies of trastuzumab-responsive epithelial CSCs (E-CSCs) and trastuzumab-refractory mesenchymal CSCs (M-CSCs). Thus, claudin-low/cHER2+ and basal/cHER2+ subtypes should contain a significant proportion of trastuzumab-unresponsive CD44^+^CD24^−/low^-expressing M-CSCs, the HER2e/cHER2+ subtype will be characterized by a high proportion of trastuzumab-sensitive ALDH-positive E-CSCs, the luminal B/cHER2 would contain a lower proportion of CSCs than HER2e/cHER2+, basal/cHER2+, and claudin-low/cHER2+ subtypes, and the luminal A/cHER2 subtype will display the lowest proportion of cells expressing any CSC marker.

### Plasticity of E- and M-CSC states and acquisition of trastuzumab-refractory M-CSC-like states

The bidirectional transition between E- and M-CSC states is mediated by plastic, EMT/MET-related epigenetic alterations contextually regulated by signals originating mostly in the tumor microenvironment (e.g., cytokines/chemokines), transcriptional regulation (e.g., microRNAs), or some combination thereof [56, 120, 127-131]. Two types of differentiated tumor cells are derived from the E- and M-CSC states: An epithelial type produced by E-CSCs, which are common in most of the cHER2+ subtypes, and mesenchymal bulk tumor cells derived from M-CSCs, which are rare in the majority of cHER2+ subtypes (i.e., luminal A/cHER2+, luminal B/cHER2+ and HER2e/cHER2+) but observable or common in the basal/cHER2+ and claudin-low/cHER2+ subtypes. While the epithelial or mesenchymal bulk cell progeny will secrete signals to positively reinforce the self-renewal of their corresponding parental E- and M-CSCs, it should be noted that more stem-like or mesenchymal phenotype cells can appear, and shift, at any time during tumor evolution, leading to mixed cell populations in cHER2+ tumors with respect to more epithelial cell properties. Thus, non-stem, bulk epithelial tumor cells in any of the cHER2+ subtypes of luminal origin can undergo an EMT phenotypic shift resulting in increased numbers of trastuzumab-refractory M-CSCs, which naturally occur at high numbers in the claudin-low/cHER2+ subtype originating from less committed mammary stem cells.

Moreover, there is evidence that more differentiated tumor cells may acquire CSC properties through nuclear reprogramming-like dedifferentiation phenomena; the range or probability of dedifferentiation rates of bulk tumor cells into CSC-like states [132-137], however, is expected to be inversely proportional to the number of cellular generations removed from being a CSC. Although all these closely related mechanisms can *de novo* repopulate CSCs in trastuzumab-treated cHER2+ BC, the intrinsic capacity to lower the epigenetic barriers responsible for cellular plasticity will vary across the molecular subtypes of cHER2+ tumors, thus determining not only their varying proportions of the two E- and M-states of CSCs, but also the proclivity to generate CSC states *via* the EMT or dedifferentiation phenomena (Figure B1-2).

### Intrinsic subtype-dependent plasticity of CSCs in cHER2+ BC

Dedifferentiation and reprogramming, two highly related versions of cancer cellular plasticity that can generate heterogeneity across the different molecular subtypes of cHER2+ BC through evolutionary time, will substantially but differentially reduce the effectiveness of trastuzumab across the spectrum of cHER2+ BC subtypes. In other words, although these mechanisms, altogether, could certainly contribute to the emergence of new clones in cHER2+ tumors with respect to plasticity for evolution and/or reversibility of tumor-initiating and self-renewal CSC-like properties, their dynamics will be intrinsically constrained not only by their inherited epigenetic programming (i.e., the cell compartments from which a given cHER2+ subtype arises), but also by the specific genetic portraits of each molecular BC subtype.

#### Epigenetic mechanisms

Chromatin state shifts may be vital to dictate plasticity in HER2+ BC cells because bivalent chromatin, i.e., simultaneous maintenance of both active and repressed marks at promoter regions of developmental genes [127-130], may hold genes in a so-called poised state that, if activated by specific microenvironmental signals, would either allow non-CSC and CSC-like mesenchymal cells to accrue epithelial characteristics, or non-CSC and CSC-like epithelial cells to accrue mesenchymal characteristics. Altered chromatin patterns during cancer development would be a factor for evolution of cellular heterogeneity [138] by helping to lock in specific tumor phenotypes. On the one hand, the development of cHER2+ BC with a luminal phenotype, which derives from committed cell compartments in normal breast epithelium, may evolve over a longer time course with more chromatin evolution from bivalency to a more stable cancer-specific promoter DNA hypermethylation. On the other hand, the development of cHER2+ BC with a claudin-low or basal phenotype, which derives from a more primitive and/or less committed normal breast epithelial compartment, might arise faster with cell phenotypes more dependent on the retention of a more plastic, epigenetically poised (bivalent) control of chromatin states.

Crucially, for our current framework of CSC-driven primary resistance to trastuzumab, such bivalent modification at specific gene promoters, which facilitate the rapid dedifferentiation of phenotypically plastic cells to stem-like cells [127-130], would permit basal-type HER2+ cells to respond to the same stimulus in a qualitatively different manner than luminal type of HER2+ cells. At least some of the non-stem, bulk epithelial cells of basal/cHER2+ will be *a priori* poised to rapidly become dedifferentiated into M-CSC-like states. Thus, in response to certain microenvironments rich in EMT-inducing heterotypic signals, basal/cHER2+ are intrinsically expected to more rapidly switch to trastuzumab-refractory M-CSC-like states than cHER2+ tumors of luminal origin. Because the gain of an increasingly stable M-CSC phenotype is apparently dependent on the sustained presence of potent EMT-reinforcing signals [127-130], in their absence, M-CSCs may naturally revert to a more epithelial phenotype unless they are supported by the appropriate epigenetic modification. Therefore, because basal/cHER2+ tumors and cHER2+ tumors of luminal origin are also differentially enriched with CD44^+^ stem cells exhibiting bivalent chromatin configurations in E-cadherin and other epithelial-specific genes, they will also exhibit an intrinsically enhanced capacity to rapidly dedifferentiate into a CD24^+^ proliferative epithelial state following trastuzumab withdrawal.

M-CSCs can stably maintain their residence in the mesenchymal state through the activation of autocrine signaling loops that liberate M-CSCs from dependence on continuous paracrine EMT-inducing signals originating elsewhere within tissues [127-130]. This scenario may naturally occur in the claudin-low/cHER2+ subtype, in which the stable residence in a mesenchymal state will involve a highly stable silencing of key epithelial genes *via* DNA hypermethylation. This can be inherited with high fidelity over the course of multiple successive divisions. Nevertheless, a full spectrum of less-plastic DNA hypermethylated to highly plastic poised states of chromatin will occur not only throughout the continuum of cHER2+ molecular subtypes but also to different extents in individual tumors, thus adding another layer of epigenetic complexity to intratumor heterogeneity in cHER2+ BC.

#### Genetic mechanisms

Beyond epigenetic diversity-mediated tolerance to trastuzumab, *bona fide* genetic mechanisms for drug resistance classically considered to solely affect the bulk cell populations might also augment CSC-driven heterogeneity in cHER2+ BC if such mutations lead to an increase in the frequency of trastuzumab-refractory M-CSC-like states. Because cHER2+ may consist of different sub-clones that carry a founder mutation(s) alone or additional acquired mutations that confer trastuzumab-resistant states, a pre-existing trastuzumab-resistant clone may remain unaffected and through outgrowth can come to dominate the entire cancer population.

For instance, *in situ* single-cell analyses are beginning to illuminate the fact that the frequency and topology of HER2 gene amplification and other key accompanying driving mutations such as PIK3CA significantly varies before and after treatment with chemotherapy. Of note, chemotherapy treatment appears to drastically modulate genetic diversity within HER2+ tumors by selecting for PIK3CA1-mutant cells, a minor subpopulation in treatment-naïve samples. Because activating mutations in PIK3CA, which are commonly associated with resistance to HER2-targeting agents [140-143], induce breast tumor heterogeneity by evoking cell dedifferentiation into multipotent stem-like states and promoting different cell fate switches [144, 145], their selection upon treatment with chemotherapeutic agents or other microenvironmental stresses might drastically accelerate tumor relapse and metastatic progression by altering the initial intrinsic phenotype of cHER2+ BC and generating M-CSC-like states refractory to anti-HER2 therapies [146]. Because mutations are shared between CSCs and their clonal progeny, the fact that intratumoral cell heterogeneity significantly increases in the spectrum of luminal-to-basal subtypes can explain how genetic and epigenetic heterogeneity can coalesce at the CSC level to differentially affect tumor evolution and clinical progression in individual tumors belonging to each cHER2+ molecular subtype (Figure B1-2).

In this integrated view, the genetic and CSC-based developmental and/or hierarchical models of BC, often considered as mutually exclusive when describing tumor heterogeneity, can be harmonized by considering the CSC state as a central biological property or epigenetic process upon which different mutational profiles across the subtypes of cHER2+ BC coalesce. Moreover, an intrinsic subtype-dependent degree of plastic, epigenetically poised (bivalent) chromatin states combined with an intrinsic subtype-dependent degree of intratumoral cell-to-cell heterogeneity may ultimately dictate how new genomic alterations acquired over time may *de novo* generate new CSCs as well as clones of differentiated progeny to differentially generate cellular heterogeneity in the natural history of individual tumors belonging to each molecular subtype of cHER2+ BC. That is to say, more poised epigenetic states in individual tumors belonging to certain subtypes of HER2+ tumor cells might generate higher degrees of diversity in gene expression patterns that can rapidly evolve through trastuzumab selection during treatment, thus driving multistep epigenetic fixation of gene expression in response to trastuzumab-based therapy.

## THERAPEUTIC IMPLICATIONS OF CSCS-RELATED INTRA-TUMOR CELL HETEROGENEITY IN CHER2+ BC

The development of specific, individualized therapeutic strategies has emphasized genomic and phenotypic differences between major BC subtypes but has largely been designed to target bulk tumor cell populations. Interestingly, the HER2-targeting antibody trastuzumab likely represents the sole currently available agent that simultaneously targets the bulk epithelial tumor population and the epithelial type of CSCs in BC. Unexpectedly, this will confound stratified treatment decisions (i.e., trastuzumab-based therapy) that are solely based on one sole mutation biomarker (i.e., HER2 gene amplification/HER2 protein overexpression). Indeed, the optimum therapeutic benefit of trastuzumab-based HER2 blockade will arise when the successful reduction of both HER2+ bulk tumor epithelial populations and HER2+-E-CSC takes place in an individual cHER2+ tumor (Figure B1-3). Conversely, poorer responses are *a priori* expected in tumors belonging to cHER2+ subtypes enriched with trastuzumab-unresponsive non-CSC mesenchymal bulk tumor cells and M-CSCs (i.e., basal/cHER2+ and claudin-low/cHER2+), or in those with an increased proclivity to alter their initial intrinsic phenotype.

**Figure B1-3:**
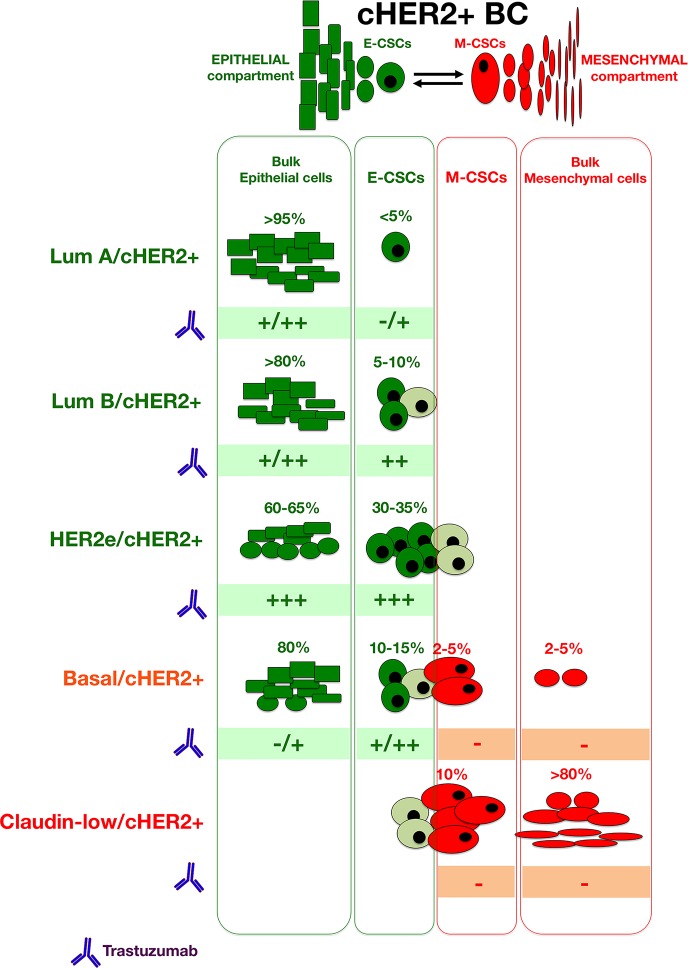
When considering the activity of trastuzumab in each type of bulk and CSC cellular compartments across the spectrum of molecular BC subtypes, we can provide a better a priori delineation of the predictive value of cHER2+ in BC in terms of trastuzumab responsiveness at the level of individual tumors, thus incorporating CSC-driven intra-tumor heterogeneity into clinical decisions
